# Investigation of Antibody Levels During Three Doses of Sinopharm/BBIBP Vaccine Inoculation

**DOI:** 10.3389/fimmu.2022.913732

**Published:** 2022-06-22

**Authors:** Jing Ma, Zhangkai J. Cheng, Mingshan Xue, Huimin Huang, Shiyun Li, Yanting Fang, Yifeng Zeng, Runpei Lin, Zhiman Liang, Huan Liang, Yijun Deng, Yuanyi Cheng, Shuangshuang Huang, Qian Wang, Xuefeng Niu, Siping Li, Peiyan Zheng, Baoqing Sun

**Affiliations:** ^1^ Department of Allergy and Clinical Immunology, State Key Laboratory of Respiratory Disease, Guangzhou Institute of Respiratory Health, National Clinical Research Center of Respiratory Disease, First Affiliated Hospital of Guangzhou Medical University, Guangzhou, China; ^2^ Clinical Laboratory, Dongguan Eighth People’s Hospital, Dongguan, China

**Keywords:** coronavirus disease 2019, vaccine, antibody persistence, booster, chemiluminescent immunoassay

## Abstract

Levels of neutralizing antibodies (NAb) after vaccine against coronavirus disease 2019 (COVID-19) can be detected using a variety of methods. A critical challenge is how to apply simple and accurate methods to assess vaccine effect. In a population inoculated with three doses of the inactivated Sinopharm/BBIBP vaccine, we assessed the performance of chemiluminescent immunoassay (CLIA) in its implementation to detect severe acute respiratory syndrome coronavirus-2 (SARS-CoV-2) specific antibodies, as well as the antibody kinetics of healthcare workers throughout the course of vaccination. The antibody levels of NAb, the receptor-binding-domain (RBD) antibodies and IgG peaked one month after the second and remained at a relatively high level for over three months after the booster injection, while IgM and IgA levels remained consistently low throughout the course of vaccination. The production of high-level neutralizing antibodies is more likely when the inoculation interval between the first two doses is within the range of one to two months, and that between the first and booster dose is within 230 days. CLIA showed excellent consistency and correlation between NAb, RBD, and IgG antibodies with the cytopathic effect (CPE) conventional virus neutralization test (VNT). Receiver operating characteristic (ROC) analysis revealed that the optimal cut-off levels of NAb, RBD and IgG were 61.77 AU/ml, 37.86 AU/ml and 4.64 AU/ml, with sensitivity of 0.833, 0.796 and 0.944, and specificity of 0.768, 0.750 and 0.625, respectively, which can be utilized as reliable indicators of COVID-19 vaccination immunity detection.

## Introduction

The SARS-CoV-2 is an extremely contagious virus that emerged unexpectedly in 2019 and remains to spread globally today (1). Spike protein (S), nucleocapsid protein (N), an envelope protein (E), and membrane protein (M) are the four structural proteins of the virus. The S protein contains different functional domains at the amino (S1) and carboxyl (S2) terminals, with S1 containing the RBD, which binds to the peptidase domain of angiotensin-converting enzyme 2 (ACE 2) to execute the first stage of infection. It is currently widely assumed that S protein, particularly S-RBD, can induce NAb (2).

Vaccination is widely acknowledged as the most effective strategy for preventing and controlling infectious diseases. Inactivated vaccinations, mRNA vaccines, and viral vaccines have all been widely utilized (3, 4). Furthermore, the inactivated vaccine has been widely used to prevent diseases caused by the influenza virus and poliovirus (5, 6), which has a high level of safety and immunogenicity. Studies have demonstrated that complete vaccination with an inactivated vaccine has been to protect against wild strains by up to 65.9% (7). Phase 1-2 clinical trials showed that the positive conversion rate of neutralizing antibodies in normal adults reached 100% 28 days after the first injection of BBIBP-CorV, and the level of neutralizing antibodies showed an upward trend within one month after the second dose (8). However, it is undeniable that its protective effect will gradually weaken over time (9), so the booster dose is essential. Prior research has indicated that neutralizing antibodies on the 14th day after the booster dose were significantly higher than those of the previous two doses (10). Meanwhile, data describing changes in antibody dynamics over time after vaccination began to emerge, but descriptions of the duration of immunity remained incomplete.

Changes in specific antibody levels (IgM, IgG, IgA) and neutralizing antibody (NAb) is not only associated with the diagnosis of the disease (11) but can also predict population immunity after vaccination (12). Currently, virus neutralization tests (VNTs) are utilized as the primary standard method for assessing neutralizing antibody levels (13), but due to their complex operations, high cost, and special facility requirements, pseudovirus neutralization tests (pVNTs) are served as substitutions for VNTs. However, neither of these two methodologies has received a widespread promotion, as the cost of performance for pVNTs is still too expensive, both in money and time, for ordinary clinics to afford (14). In contrast, CLIA provides high-throughput, rapid, and reproducible assays for neutralizing antibodies, as well as detecting trends and influencing variables in IgM, IgG, IgA, RBD, and neutralizing antibody levels after vaccination. As a result, the objective of this study is to investigate the changes in antibody levels after inactivated vaccine vaccination as well as evaluate the detection performance of CLIA, providing a valuable reference for vaccine development, drug therapy, epidemiology, and immune monitoring.

## Materials and Methods

### Study Subjects

Blood samples were taken from healthcare workers who had received inactivated vaccinations at the First Affiliated Hospital of Guangzhou Medical University between December 27, 2020 and January 13, 2022. The study protocol was approved by the Ethics Committee of the First Affiliated Hospital of Guangzhou Medical University (2021, No.31) and informed consent was obtained from each volunteer. The vaccine was inoculated three times, 0.5mL each, using Sinopharm/BBIBP (Beijing Bio-Institute of Biological Products Co., Ltd.) vaccine. The recommended interval between the first and second doses is 21-28 days, and the booster dose is recommended to be completed within 6 months of the first dose. Before the immunization (V1), the second dose (V2), 7 days after the second dose (V2+7), one month after the second dose (V2+30), and two months after the second dose (V2+60), 5 months after the second dose (V2+150), 14 days after the booster dose (180 V3+14), one month after the booster dose (180 V3+30) and three months after the booster dose (180 V3+90), a total of nine time points were followed up and samples were collected. After standing for two hours, all the whole blood samples were separated at 1600 rpm/s for 10min under sterile conditions and stored at -80°C. Serum samples were used for VNTs, while plasma samples were used for CLIA and pVNT. All plasma samples were mixed by freeze-thawing before detection, and the supernatant was obtained for detection after 10000 rpm/s within 10 min.

Inclusion criteria: healthy healthcare workers who signed an informed consent form, all volunteers had a negative nucleic acid test during the monitoring period and had no contact history with suspected or confirmed cases of COVID-19. Exclusion criteria: severe allergic reactions to previous vaccinations or allergic reactions to active ingredients, inactive ingredients or substances used in the preparation of vaccines contained in the new crown vaccine, and allergic reactions after the first dose; patients with severe chronic diseases or in the acute exacerbation stage of chronic diseases; patients with platelet dysfunction or bleeding disorder; patients with severe immunodeficiency diseases, liver and kidney diseases, malignant tumors, etc.; in the period of other vaccinations.

### CPE Neutralization Test Based on SARS-CoV-2 Live Virus Cells

All serum samples were labeled and inactivated at 56°C for 30 minutes before being examined. 180 μl of maintenance solution was added to each well on a 96-well plate. 60 μl of the heat-inactivated serum was added to the first well, then 60 μl of the diluted serum was transferred to the next row and mixed evenly. 1:4 gradient dilution was performed 5 times, and the excess 60 μl of liquid was discarded from the last row.

125 μl of each dilution was transferred to a separate 96-well plate. The SARS-CoV-2 virus was diluted to 100 TCID50/50 μl using Dulbecco’s Modified Eagle’s culture medium (DMEM) +2% fetal bovine serum (FBS). The diluted virus was added at a ratio of 1:1 (V/V), then the virus was placed in an incubator with 5% CO2 at 36°C for 2 hours after being shaken evenly and gently once after culture for 1.5 hours. VeroE6 cells were inoculated at a density of 1×10^4^~2×10^4^ cells per well into 96-well plates. After neutralization, 100 μl of VeroE6 cells were added to the cell hole and cultured in a 36°C incubator with 5% CO_2_. CPE in each well was recorded on days 5-8 when complete lesions formed in the 100 TCID50 antigen control, with the results monitored every day. The SARS-CoV-2 neutralizing antibody titer of the serum is the reciprocal of the maximum serum dilution that can protect 50% of cell wells from CPE.

### Neutralization Test Based on SARS-COV-2 Pseudovirus

To manufacture SARS-CoV-2 pseudovirus, the SARS-CoV-2 spike expression plasmid was cotransfected into HEK-293t cells with an HIV-1 backbone vector containing a luciferase reporter gene. The virus was diluted to 1-2 × 10^4^ TCID50/ml in DMEM medium containing 10% FBS serum. The plasma after heat extinguishment was diluted 20 times with DMEM medium and filtered *via* a 0.22 μm filter membrane. Diluted 30 times in the first well, mixed evenly, then diluted three times in six gradients, and discard the excess 50 μl in the last row. Incubate at 37°C for one hour with 50 μl of diluted virus in each well. Add 50 μl 293-ACE2 with a density of 0.4 × 10^6^ cells/ml to each well, culture at 37°C with 5% CO2 for 48 h, then take out the 96-well whiteboard, equilibrate to room temperature, suck out 100 μl medium from the well plate, add 100 μl Bio-Lite reporter reagent after equilibrating to room temperature. The plate was shaken for 2 min and placed at room temperature for 5 min. The relative light unit (RLU) was detected by a microplate reader.

### Chemiluminescence Immunoassay

The SARS-CoV-2 NAb testing employs CLIA (competitive method). The detection principle utilizes the idea that the NAb of SARS-CoV-2 will impede ACE2’s particular binding to RBD. Magnetic particles were coated with recombinant ACE2 antigen and labeled with recombinant RBD antigen by horseradish peroxidase. The RBD antigen, which was not neutralized by antibodies, formed a complex with ACE2 on magnetic particles. These complexes catalyze the emission of photons from luminous substrates, the intensity of which is inversely proportional to the concentration of the antibody. When the concentration value of the sample is less than 30.00 AU/ml, it is regarded as positive for neutralizing antibodies of wild strain. When a sample’s concentration value is less than 30.00 AU/ml, it is regarded as negative.

The capture method was used to detect SARS-CoV-2 IgM, anti-human IGM was coated with magnetic particles, and horseradish-peroxidase-labeled SARS-CoV-2 antigen to prepare enzyme conjugate. SARS-CoV-2 IgG and IgA were detected by indirect method, using SARS-CoV-2 antigen-coated with magnetic particles and horseradish-peroxidase-labeled anti-human IgG/IgA antibody to form solid-phase antigen-IgG/IgA antibody-conjugated secondary antibody complex. The sandwich method was used to detect anti-SARS-CoV-2 RBD antibody, using SARS-CoV-2 RBD antigen coated with magnetic particles, and horseradish-peroxidase-labeled SARS-CoV-2 RBD antigen to form solid-phase SARS-CoV-2 RBD antigen-specific antibody-enzyme conjugate antigen complex. These complexes catalyze the emission of photons from luminous substrates, whose intensity is proportional to antibody concentration. The manufacturer-defined positive threshold for IgM, IgG, and IgA were all 1 AU/ml, while the positive threshold for RBD was 8 AU/ml. Autolumo A2000 PLUS Automatic Chemiluminescence immunoassay (Autobio Diagnostics Co., Ltd.) was used as a matching instrument for the above reagents.

### Statistical Analysis

SPSS 25.0 was used for analysis. The Mann-Whitney U test was used to assess the measurement data between the two groups, and median and quartile distance were used to describe them. The Spearman correlation analysis was used to analyze correlation between two groups. *P ≤* 0.05 was considered statistically significant. Graph Pad Prism 8.0 (La Jolla, USA) was used to plot the results.

## Results

### Baseline Data

**Figure 1:**
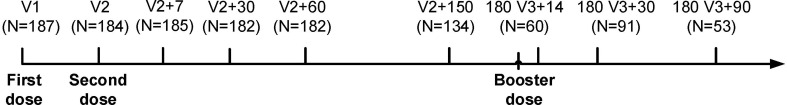
Group entry process and time interval. V1: first injection (before immunization). V2: second injection. V2+7: seven days after the second injection. V2+30: one month after the second injection. V2+60: two months after the second injection. V2+150: five months after the second injection. 180 V3+14: 14 days after booster injection. 180 V3+30: one month after booster injection. 180 V3+90: three months after booster injection.

187 participants were recruited from the First Affiliated Hospital of Guangzhou Medical University, with none having a prior history of COVID-19 and were negative for SARS-COV-2 nucleic acid tests, due to the Zero-COVID strategy employed in China (15). The basic information of participants is shown in **Table 1**, which includes 39 males and 148 females, with a male to female ratio of 1:3.79. The participants ranged in age from 20 to 58 years old, with an average age of 37.1 years. Blood samples were obtained at various time intervals to monitor the dynamic changes in antibody levels in individuals, as depicted in **Figure 1** and **Supplementary Table 1**.

**Table 1:** Baseline characteristics.

		N	Mean ± SD/%
Sex	Male	39	20.86%
	Female	148	79.14%
Age (years)			37.06 ± 8.76
	20-29	45	24.07%
	30-39	72	38.50%
	40-49	57	30.48%
	≥50	13	6.95%

### Plasma Antibody Kinetics Throughout BBIBP Vaccination

**Figure 2:**
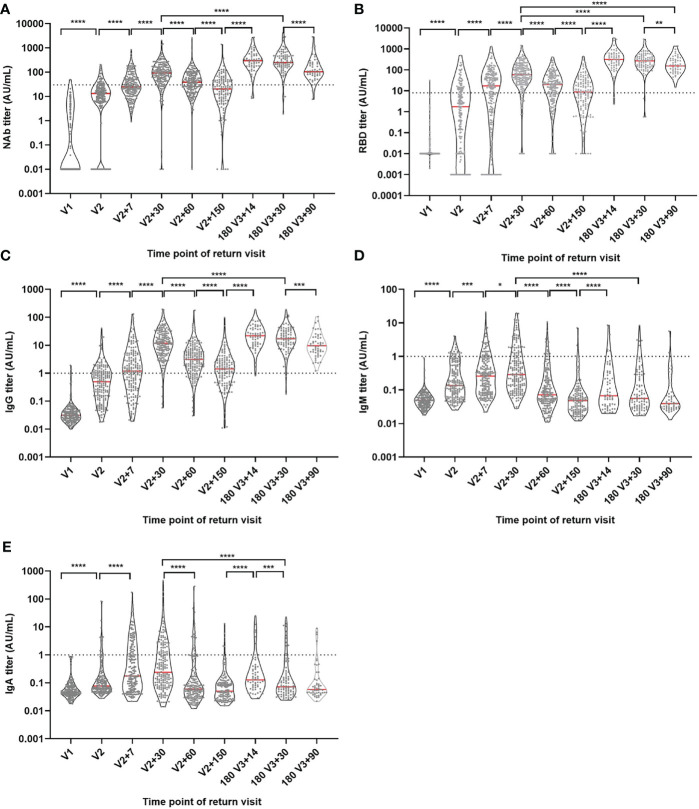
Antibody titer levels at different time points during vaccination. **(A)** NAb antibody level. **(B)** RBD antibody level. **(C)** IgG antibody level. **(D)** IgM antibody level. **(E)** IgA antibody level. Red lines: median titer. Dotted lines: manufacturer-defined positive threshold values. *P ≤ 0.05. **P ≤ 0.01. ***P ≤ 0.001. ****P ≤ 0.0001 (Mann-Whitney U test).

CLIA was used to examine the development of IgG, IgA, IgM, RBD, and NAb antibodies during immunization (**Figure 2**). We found that NAb, RBD and IgG had a consistent trend of change, and the antibody level showed an increasing trend after the first dose, and further increased after the second dose and reached the highest level (peak) one month after the second dose, then slowly decreased. The levels of NAb, RBD and IgG antibodies were further increased after the booster dose and the antibody levels were significantly decreased but remained high three months after the booster dose, even the antibody levels of RBD were significantly higher than one month after the second dose (P<0.05). However, IgA and IgM also reached their peak one month after the second injection. Although IgA and IgM levels were significantly increased after the booster injection, the peak antibody level was significantly lower than that one month after the second dose (P<0.05). We also looked at age (20-29, 30-39, 40-49 and ≥50 years old) and gender groups, the results showed that there was no significant difference in the trend of antibody levels among different ages and genders (**Supplementary Figures 1, 2**).

**Figure 3:**
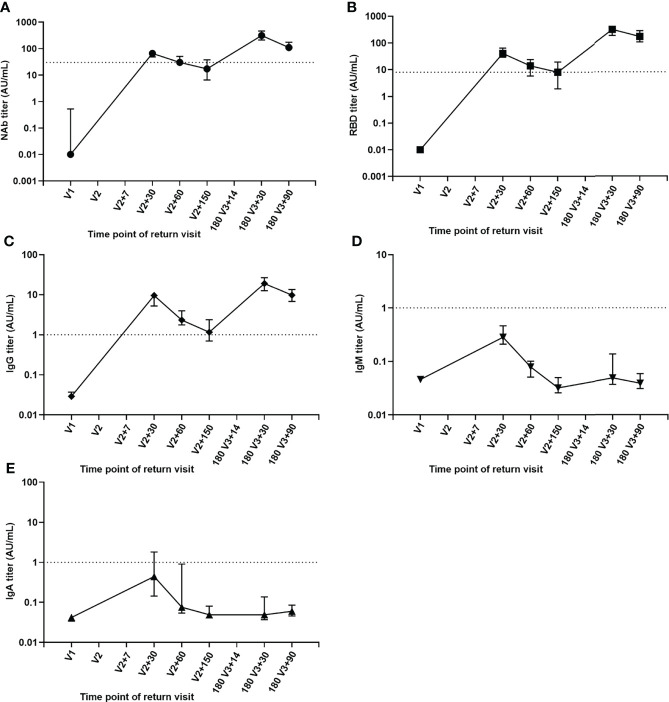
Kinetics of antibody levels for sequential samples. **(A)** NAb antibody. **(B)** RBD antibody. **(C)** IgG antibody. **(D)** IgM antibody. **(E)** IgA antibody.

A total of 37 volunteers, with a male to female ratio of 1:2.7 and an average age of 39.08 ± 8.44, were selected for continuous follow-up at six main time points: V1, V2+30, V2+60, V2+150,180 V3+30 and 180 V3+90 and the dynamic changes of their antibody levels were analyzed. Compared with one month after the second injection, NAb, RBD and IgG antibody titer levels were 3.8, 5 and 8.2 times lower at 5 months after the second dose (**Figures 3A–C**), but increased by 4.7, 7.6 and 2 times on one month after the booster dose. Compared with one month after the booster injection, the median level of NAb, RBD and IgG antibodies decreased by 2.8 times, 1.8 times and 2 times three months after the booster dose. Despite the fact that antibody levels were much lower three months after the booster dose, the median NAb and RBD antibody titer levels were still 1.6 and 4.1 times higher than they were one month after the second dose. Meanwhile, we observed that one month after the second dose, the peaks of IgM and IgA antibody titer levels were attained (**Figures 3D, E**) but the booster dose did not significantly increase their titer levels.

### Effects of Different Inoculation Intervals on Antibody Levels

**Figure 4:**
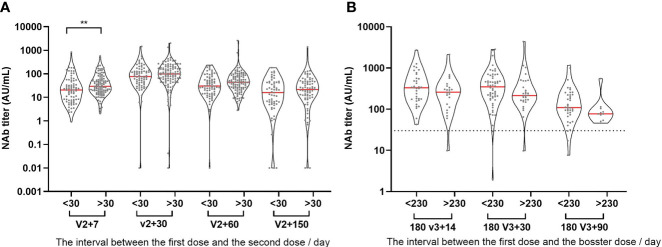
Effects of different inoculation intervals on antibody levels. **(A)** The relationship between NAb titer level and the time interval for the first two injections. **(B)** The relationship between NAb titer level and the time interval for the first and booster injection. >30: the interval between the first two injections is longer than or equal to 30 days. <30: the interval between the first two injections is less than 30 days. >230: the interval between the first and booster injection is longer than or equal to 230 days. <230: the interval between the first and booster injection is less than 230 days. **P ≤ 0.01 (Mann-Whitney U test).

Vaccine administration at regular intervals is difficult in the actual world. As a result, the time interval between the first dose and the second dose is wide in actuality. The currently recommended time interval for inactivated vaccinations is one month, with 30 days serving as the dividing line. The longer the interval between the first and second doses (25-57 days in this study), the higher the NAb titer levels produced (**Figure 4A**). The interval between the first and booster doses in our study was 200-276 days. NAb titer levels after the booster injection were compared at the median level of 230 days as the dividing line. It was discovered that a longer time interval did not increase the antibody level after three doses, but rather had a declining tendency (**Figure 4B**).

### Performance Verification of Neutralizing Antibody Detection by Chemiluminescence Immunoassay

**Figure 5:**
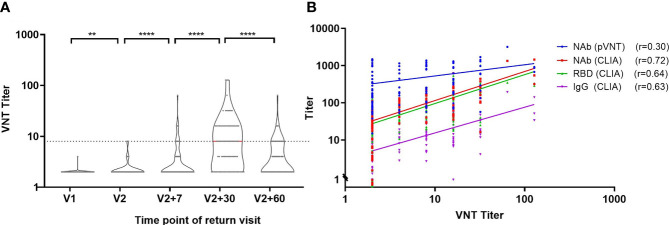
Correlation analysis of CLIA, VNT and pVNT antibody detection. **(A)** Trend of VNT titer over time. **(B)** Correlation between neutralizing antibodies detected by VNT and pVNT and NAb, RBD and IgG antibodies detected by CLIA. r: Spearman correlation coefficient. **P ≤ 0.01. ****P ≤ 0.0001 (Mann-Whitney U test).

We validated neutralizing antibody levels using VNT and pVNT in addition to the CLIA. The trends of NAb, RBD and IgG antibodies were consistent with those of VNT titers, and the first peak of antibody levels appeared one month after the second injection (**Figure 5A**). In order to perform comparison between the CLIA, VNT and pVNT methods, we analyzed 111 samples during follow-up that were simultaneously detected with all three methods and found that the NAb, RBD and IgG antibodies have strong correlations with VNT titers (**Figure 5B**), with correlation coefficients of 0.72, 0.64 and 0.63, respectively. On the other hand, the correlation between pVNT and VNT results have a weak correlation, having a correlation coefficient of only 0.30.

**Figure 6:**
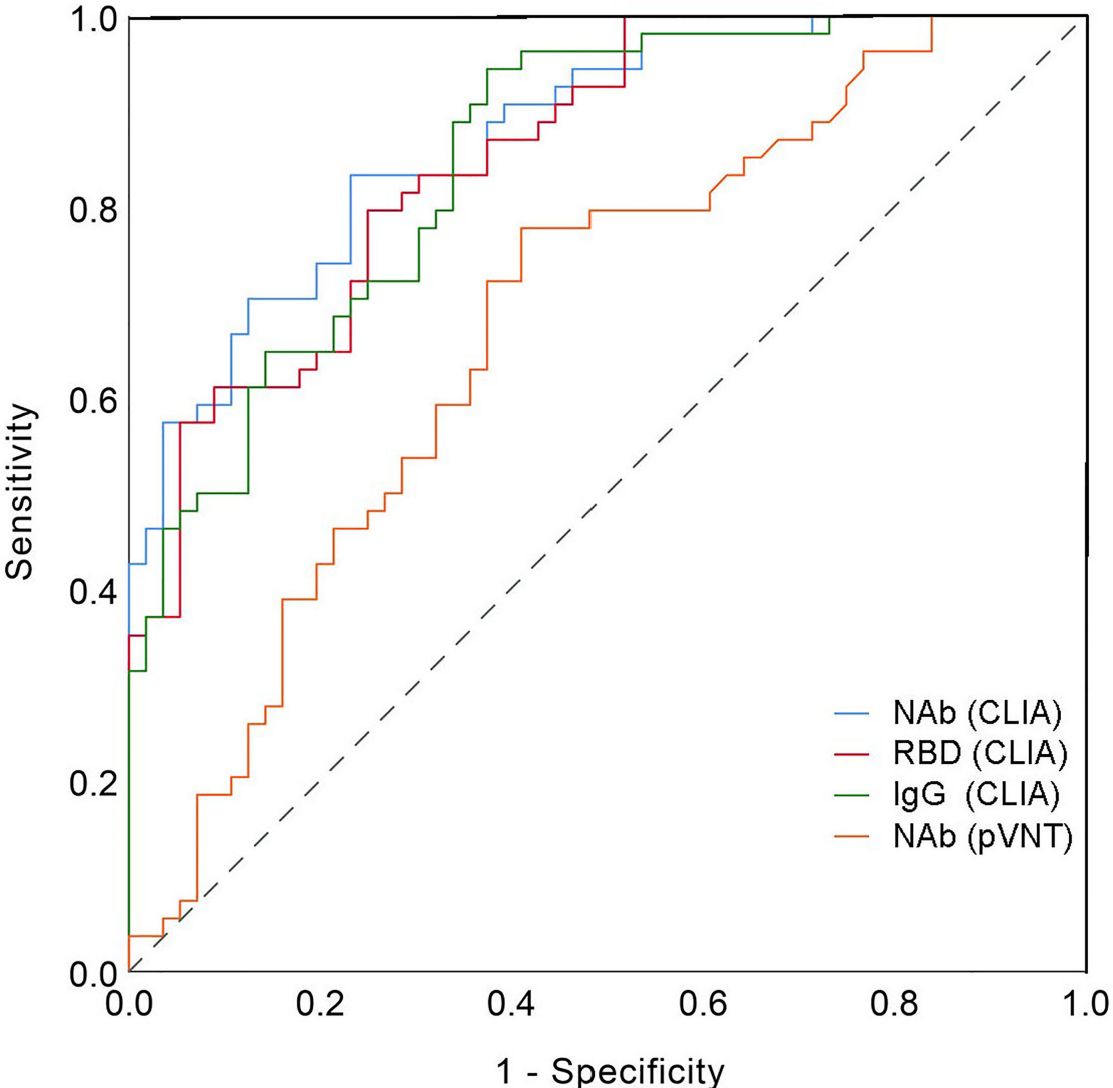
Receiver operating curve (ROC).

Then, using VNT titers as the standard, the optimal cut-off values of NAb, RRD, IgG and pVNT titers by receiver operating characteristic (ROC) curve were calculated to be 61.77 AU/mL, 37.86 AU/mL, 4.64 AU/mL and 182 (ID50), respectively (**Table 2**). The area under curve (AUC) were 0.875, 0.855, 0.852, and 0.681, respectively. At this cut-off value, NAb, RBD and IgG all had good detection performance and were superior to pVNT methodology (**Figure 6** and **Table 2**).

**Table 2:** Optimal cut-off values.

	AUC	95%CI	Cutoff	Sensitivity	Specificity	Youden index
NAb	0.875	0.812-0.937	61.77	0.833	0.768	0.601
RBD	0.855	0.788-0.922	37.86	0.796	0.750	0.546
IgG	0.852	0.783-0.920	4.64	0.944	0.625	0.569
pVNT	0.681	0.581-0.781	182.00	0.778	0.589	0.367

## Discussion

Since the prevalence of COVID-19 from early 2020 to the present, vaccines are considered the primary way to defend against the viruses. As a result, various types of vaccines swiftly enter the clinic and are widely administered, such as inactivated virus vaccines, mRNA vaccines encoding the spike protein of SARS-CoV-2, and viral vector-based vaccines (e.g. adenovirus). The inactivated vaccine is undoubtedly the earliest and most widely used vaccine in China. The two whole virus inactivated vaccines provided 72.8% and 78.1% protection in clinical studies of healthy individuals injected with domestic inactivated vaccines (16). Although the protection rate is lower than that of some RNA and adenovirus vaccines (17, 18), the advantages of inactivated vaccines in large-scale and diverse populations are determined by their superior safety and immunogenicity.

The neutralizing antibody is undoubtedly the best choice for monitoring vaccine protection, and their detection results are subject to VNT and pVNT. However, the complexity of the procedure and the length of time it takes to complete limit its widespread application (14). Relatively, CLIA is a rapid and stable method, which is widely used in the early stage of COVID-19 outbreak (19). The combination of IgG and IgM detection is crucial in the diagnosis of COVID-19. In addition, the CLIA also provides the possibility to replace the conventional standard method in the monitoring of the immune effect of vaccines (20, 21). CLIA was primarily used in our study to detect changes in various antibody levels after inactivated vaccine inoculation. According to our comparison, the variation trend of VNT and pVNT was consistent with the results of NAb, RBD and IgG detected by the CLIA, and there was a significant association between NAb, RBD and IgG and VNT results. In comparison to VNT, NAb detection has a sensitivity of up to 63%, but its specificity is low. ROC curves were used to determine the optimal thresholds for NAb, RBD, IgG and pVNT and they were 61.77 AU/ml, 37.86 AU/ml, 4.64 AU/ml and 182 (ID50). The sensitivity was 0.833, 0.796, 0.944, and 0.778 under the cutoff value, while the specificity was 0.768, 0.750, 0.625, and 0.589. According to studies, the sensitivity of NAb, RBD, and IgG antibody detection by CLIA is no less than pVNT, and the specificity is even better. In general, CLIA can be used as a reliable indicator of vaccine immunity detection.

The dynamic variations in antibody levels after vaccination are critical for monitoring population immunity and preventing viral infection. The trajectory of antibody alterations in the population revealed that the highest antibody level peaked in the first month after the second dose. Related studies have found that antibodies generated by the inactivated vaccine can still be detected in humans up to month 6 after the second dose (22). We also discovered that NAb detection could still be positive in some populations five months after the second dose. Compared to one month after the second dose, NAb, RBD, and IgG antibody titer levels declined approximately 3.8, 5, and 8.2 times, respectively, whereas IgA and IgM antibody levels reduced around 9 times. Similar to inactivated vaccine antibody levels, BNT162b2 (Pfizer-BioNTech) vaccine antibody levels peaked one month after vaccination (23), while mRNA-1273 peaked around 2 weeks after the second dose and subsequently decreased (24). At 6 months after the second dose, antibody levels of the BNT162b2 vaccine were approximately 2-fold lower (23) and neutralizing antibody levels of mRNA-1273 were approximately 4-fold lower (24). However, due to the heterogeneity of the antibody neutralization analysis, it is difficult to directly compare these estimates with the results of this study. A month after a booster injection, the antibody levels began to decline significantly. At three months after the booster dose, median levels of NAb, RBD, and IgG antibodies were 2.8, 1.8 and 2 times lower than the first month after the booster. However, the median levels of NAb and RBD antibodies in the third month after the booster dose were still 1.6 times and 4.1 times higher than those in the first month after the second dose, while the levels of IgA and IgM antibodies were about 1/7 of those in the first month after the second dose. In conclusion, NAb, RBD and IgG antibody levels were significantly decreased by the third dose of the vaccine. It has remained at a high level for three months, which is higher than the first month after the second dose.

In our study, the levels of IgA and IgM were consistently low, and their antibody levels were not further boosted by the booster vaccination. The researchers discovered that detectable IgM levels were typically lower after vaccination and lasted for a shorter period of time, as a result, IgM may be of minor consequence in viral neutralization *in vivo* (25, 26). Mucosal immunity is a critical aspect in preventing and resisting infection in SARS-CoV-2, which is a respiratory transmission virus (27). Plasma IgA levels not only correlate with mucosal IgA levels (28) but also exhibit a higher neutralization capacity early in infection (29, 30). Only 1.7 percent of participants remained IgA negative against S1 in the first month after the second dose in related investigations using mRNA vaccine (31), which was significantly higher than the IgA positive rate induced by inactivated vaccination. Furthermore, we discovered that IgA and IgG had identical induction kinetics and times to peak levels during the first two doses vaccinations (32). We further observed that the booster dose vaccination does not further improve the level of IgA. Moreover, relevant investigations revealed that the development of specific antibody IgA occurred earlier than IgG after inactivated vaccine immunization (33). However, we did not observe this trend due to the low levels of IgA in plasma or discrepancies in methodological detection.

Vaccine effectiveness is affected by a variety of elements, including the underlying disease of the vaccinee, age, gender, and the time interval between vaccine booster doses (22, 28, 34–36). Previous research has demonstrated that underlying illnesses can alter antibody levels before immunization (28, 35, 36). Male populations beyond the age of 65 will have significantly lower levels of antibodies (22, 36). The longer the time interval between the first and second dose, the more favorable is the production of high levels of antibodies (7). Our study found that there was no significant difference in the trend of antibody change in people under 58 years old, and gender was not related to the change in antibody level. We also discovered that a time interval of one to two months between the first and second doses, while less than 230 days between the first and booster doses were more conducive to the production of high levels of neutralizing antibodies in our study. Our research involved a group of healthcare workers, among this cohort contains a higher proportion of women, most of whom are healthy and young, therefore the study group does not represent the general public. However, this cohort is a highly exposed and high-risk population, and assessing their vaccination protection is clinically meaningful.

In conclusion, our study found that chemiluminescence NAb, RBD and IgG antibodies can be reliable indicators for vaccine immunity detection. People who received three doses of inactivated vaccine declined after peaking one month after the second dose, and antibody levels persisted at higher levels for more than three months after the booster dose. Furthermore, the optimal interval for the second dose is one to two months, while the optimal interval for the booster dose is 230 days.

## Data Availability Statement

The original contributions presented in the study are included in the article material, further inquiries can be directed to the corresponding authors.

## Ethics Statement

The studies involving human participants were reviewed and approved by The First Affiliated Hospital of Guangzhou Medical University Scientific Research Project Reviews Ethics Committee, clinical research approval 2021 No.31. The patients/participants provided their written informed consent to participate in this study.

## Author Contributions

All authors made a significant contribution to the work reported, whether that is in the conception, study design, execution, acquisition of data, analysis, and interpretation, or in all these areas; took part in drafting, revising, or critically reviewing the article; gave final approval of the version to be published; have agreed on the journal to which the article has been submitted; and agree to be accountable for all aspects of the work.

## Funding

This study was supported by the emergency key project of Guangzhou Laboratory (EKPG21-30-2), Zhongnanshan Medical Foundation of Guangdong Province (ZNSA-2021005), State Key Laboratory of Respiratory Disease, Guangdong-Hong Kong-Macao Joint Laboratory of Respiratory Infectious Disease (GHMJLRID-Z-202102). Cultivation Project of the First Affiliated Hospital of Guangzhou Medical University (ZH202105), and Guangzhou Institute of Respiratory Health Open Project (Funds provided by China Evergrande Group) (2020GIRHHMS04).

## Conflict of Interest

The authors declare that the research was conducted in the absence of any commercial or financial relationships that could be construed as a potential conflict of interest.

## Publisher’s Note

All claims expressed in this article are solely those of the authors and do not necessarily represent those of their affiliated organizations, or those of the publisher, the editors and the reviewers. Any product that may be evaluated in this article, or claim that may be made by its manufacturer, is not guaranteed or endorsed by the publisher.
