# Supplementary material for: Investigation of Antibody Levels During Three Doses of Sinopharm/BBIBP Vaccine Inoculation
Source: Front Immunol. 2022 Jun 22;13:913732. doi: 10.3389/fimmu.2022.913732 (PMC9256989; doi:10.3389/fimmu.2022.913732)
Supplement: Supplementary file 1 [file DataSheet_1.docx]

Supplementary Table 1: Return time interval

| **Time point of return visit** | | **N** | **Interval with the first dose/day** |
| --- | --- | --- | --- |
| V1 |  | 187 | 0 |
| V2 |  | 184 | 33.31±5.78 |
| V2+7 |  | 185 | 40.70±6.20 |
| V2+30 |  | 182 | 62.31±6.55 |
| V2+60 |  | 182 | 125.37±10.59 |
| V2+150 |  | 134 | 188..62±9.32 |
| 180 V3+14 |  | 60 | 240.85±11.79 |
| 180 V3+30 |  | 91 | 259.17±14.17 |
| 180 V3+90 |  | 53 | 317.59±7.38 |


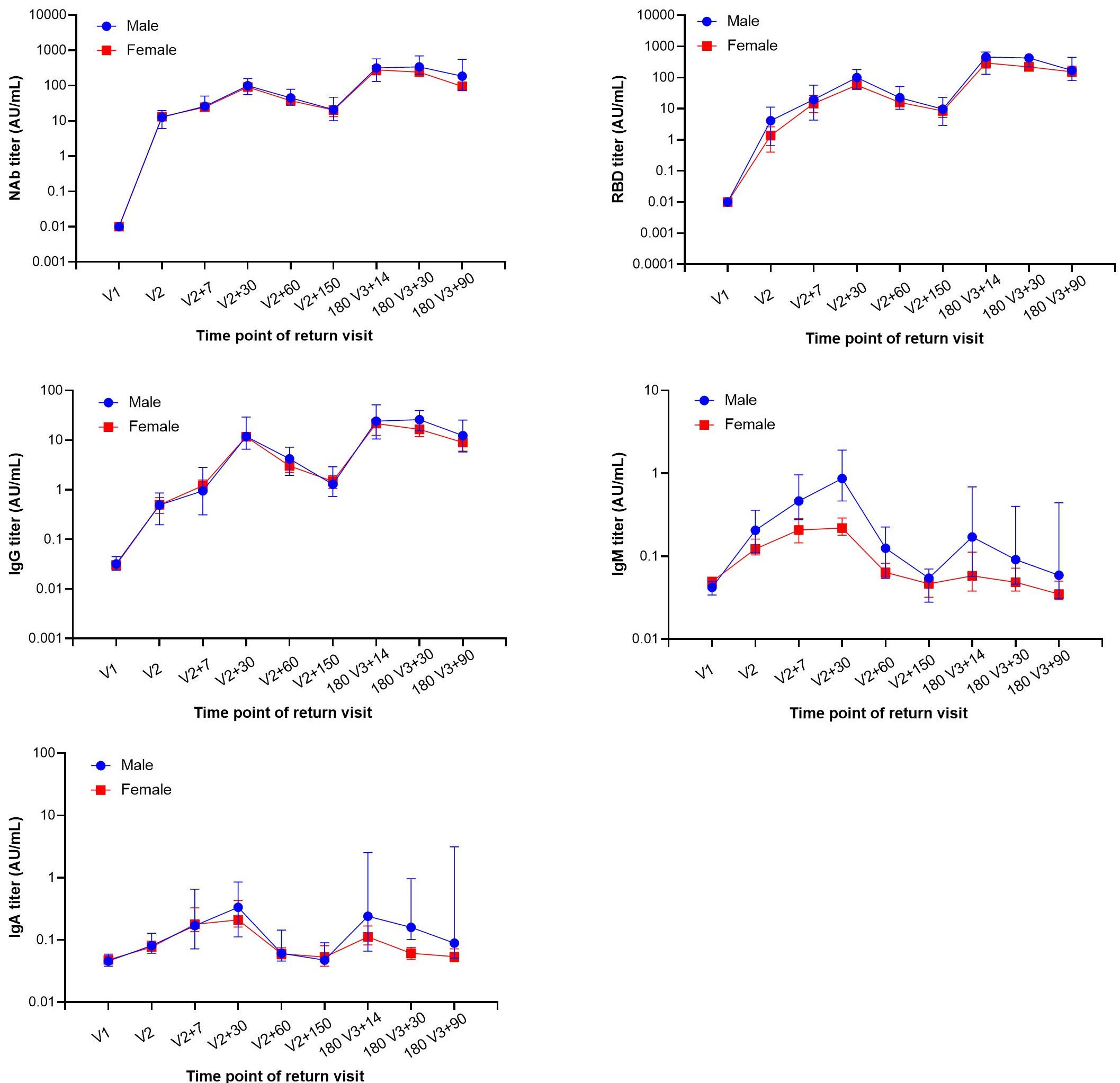


**Supplementary figure 1. The antibody titer time profile for the two genders.** (A) NAb antibody. (B) RBD antibody. (C) IgG antibody. (D) IgM antibody. (E) IgA antibody.


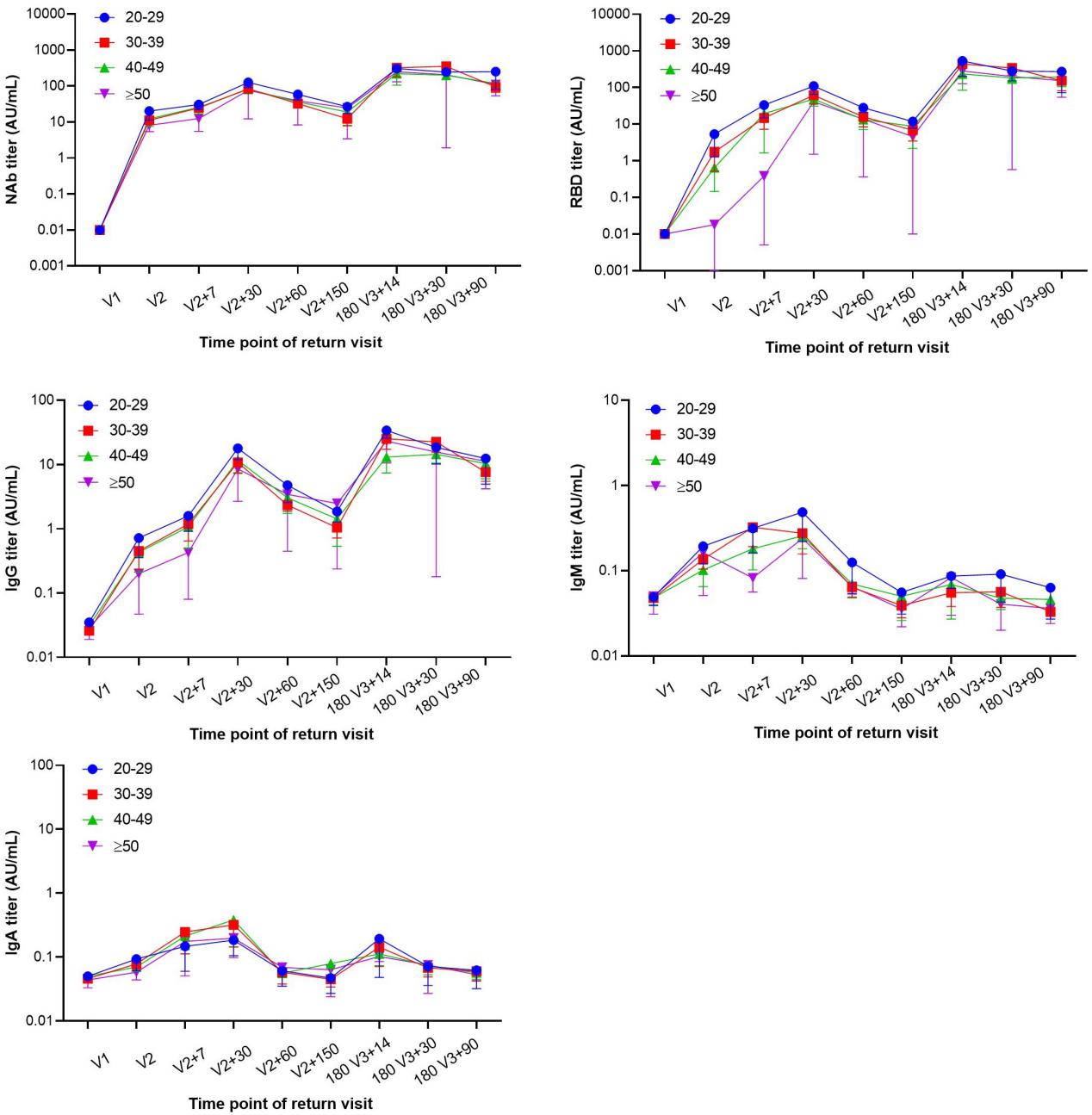


**Supplementary figure 2. The antibody titer time profile for the four age groups (20-29, 30-39, 40-49 and ≥50 years old).** (A) NAb antibody. (B) RBD antibody. (C) IgG antibody. (D) IgM antibody. (E) IgA antibody.
